# Translation, cross‐cultural validity and reliability of a Danish version of the Banff Patella Instability Instrument 2.0 (BPII 2.0)

**DOI:** 10.1002/jeo2.70649

**Published:** 2026-01-19

**Authors:** Torsten Grønbech Nielsen, Martin Lind, Simon Damgaard Petersen, Pia Kjær Kristensen

**Affiliations:** ^1^ Sports Traumatology, Orthopedic Department Aarhus University Hospital Aarhus N Denmark; ^2^ Department of Physiotherapy and Occupational Therapy Aarhus University Hospital Aarhus N Denmark; ^3^ Department of Clinical Medicine Aarhus University Aarhus N Denmark; ^4^ Department of Orthopedic Kolding Hospital Kolding Denmark

**Keywords:** Banff Patella Instability Instrument 2.0 (BPII 2.0), outcome measure, patellofemoral instability, translation, validation

## Abstract

**Purpose:**

This study aimed to translate and cross‐culturally validate a Danish version of the BPII 2.0, and assess its construct validity, internal consistency and test‐retest reliability in a Danish population of patients with patellar instability.

**Methods:**

The BPII 2.0 was translated and cross‐culturally validated according to international guidelines, including 15 think‐aloud interviews in patients with patellar instability. Responses from 100 patients with patellar instability were accustomed to evaluating, construct validity, floor and ceiling effect, minimal detectable change, internal consistency and test‐retest reliability. Construct validity was assessed using Spearman Rho (*r*) comparing the BPII 2.0‐DK with the Kujala‐DK, the International Knee Documentation Committee (IKDC), the Victorian Institute of Sport Assessment‐Patella (VISA‐P) and the Tegner activity score (TAS). Structural validity was assessed using confirmatory factor analysis (CFA). Internal consistency reliability was evaluated using Cronbach's alpha. Test‐rest reliability was examined using Pearson's intraclass correlation coefficient (ICC) in 57 patients who completed the BPII 2.0 twice, with a one‐week interval. The study followed the COSMIN guidelines.

**Results:**

Minor comprehensibility issues were corrected during translation, and patients found the BPII 2.0‐DK relevant and comprehensive. High positive correlations were found with the Kujala‐DK (*r* = 0.73), IKDC (*r* = 0.81) and VISA‐P (*r* = 0.67), whereas only a low correlation was found with the TAS (*r* = 0.44). CFA demonstrated loadings ranging from 0.45 to 0.84. No ceiling or floor effects were observed, and a minimal detectable change of 15.3 points was found. Cronbach's alpha was 0.95, indicating excellent internal consistency, and ICC was 0.90 (95% CI: 0.86–0.94) for test‐retest reliability.

**Conclusion:**

The BPII 2.0‐DK is a relevant, comprehensible, and comprehensive tool for patients with patellar instability. There was a high positive correlation between BPII 2.0 and Kujala‐DK, IKDC and VISA‐P. Furthermore, excellent internal consistency reliability and high test‐retest reliability were found.

**Level of Evidence:**

Level III.

**Abbreviations**
AAOS: American Association of Orthopedic Surgeons
BPII 2.0: Banff Patella Instability Instrument
CFA: confirmatory factor analysis
CI: confidence intervals
COSMIN: consensus‐based standards for the selection of health measurement instruments
ICC: interclass correlation coefficient
IKDC: International Knee Documentation Committee
Kujala: Anterior Knee Pain Scale
LoA: limits of agreement
MDC: minimal detectable change
MPFL: medial patella femoral ligament
NPI: Norwich Patellar Instability
PROM: patient‐reported outcome measures
SD: standard deviation
SEM: standard error of measurement
TAS: Tegner activity score
VISA‐P: Victorian Institute of Sports Assessment—Patella questionnaire

## INTRODUCTION

Patellar instability is a disabling condition primarily affecting young, active individuals, which may lead to recurrent dislocations, pain, and an overall decrease in quality of life [15]. Patient‐reported outcome measures (PROMs) are essential for evaluating the health status and treatment effectiveness [5]. Currently, the Banff Patellar Instability Instrument 2.0 (BPII 2.0) and the Norwich Patellar Instability (NPI) questionnaire are the only PROMs that have been specifically developed for patients with patellar instability [10, 11, 15, 18]. Of these, the BPII 2.0 is the most widely used [18]. The original BPII was developed in 2013 to assess the quality of life in adults following patellar dislocation [10]. In 2016, the instrument underwent revision based on factor analysis, resulting in a shorter, 23‐item version: the BPII 2.0 [11]. Since then, this version has undergone cross‐cultural validation in German, Indonesian, Norwegian, Swedish, Turkish, Spanish and Arabic gaining international recognition as a disease‐specific PROM for patients with patellar instability [1, 4, 12, 19, 24, 29, 30]. Despite the widespread use of PROMs in Danish orthopaedic research and clinical practice [8, 16, 17], no Danish version of the BPII 2.0 currently exists. Instead, the anterior knee pain scale (Kujala score) has been used more commonly in Danish patients with patellar instability, even though it was originally developed for patients with patellofemoral pain [2].

The psychometric properties of the BPII 2.0 have been investigated in patients with patellar instability, except for the Indonesian version, which was tested on a population with patellofemoral pain [1, 4, 12, 19, 24, 29, 30]. These studies have generally supported the construct validity, internal consistency and test‐retest reliability of the instrument. Five studies included the Kujala score as a comparator and reported Pearson correlations ranging from 0.53 to 0.84 in populations with patellar instability. Notably, the Indonesian version was tested on a population with patellofemoral pain, yielding an unusually high correlation (*r* = 0.98), raising questions about the appropriateness of the sample. While these studies offer promising psychometric evidence, five out of seven included fewer than 65 participants, which falls short of the minimum sample size of 100 recommended by the COSMIN (Consensus‐Based Standards for the Selection of Health Measurement Instruments) guidelines for a robust psychometric assessment [20]. The Norwegian study is the only exception, as it had a sufficient sample size. However, it relied on comparator instruments that had not been validated in Norwegian [12]. A Danish validation therefore serves two purposes: first, it extends the cross‐cultural body of evidence by applying internationally accepted psychometric standards in a new linguistic and clinical setting, and introduces confirmatory factor analysis (CFA) to assess the structural validity of the BPII 2.0, a method not previously utilised in earlier studies; secondly, it ensures that the future use of the BPII 2.0 in Denmark is based on methodologically sound and culturally appropriate foundation.

## AIM

The objective was therefore to translate and cross‐culturally adapt the BPII 2.0 into Danish, and to test its psychometric properties—construct validity, internal consistency, and test‐retest reliability—in a consecutive cohort of Danish patients with patellar instability.

## MATERIALS AND METHODS

### BPII 2.0

The BPII 2.0 was developed to assess the quality of life of patients with patellar dislocation from a holistic perspective [10]. This 23‐item questionnaire uses a visual analogue scale ranging from 0 to 100. It consists of five domains: ‘symptoms and physical complaints’, ‘work and/or school related concerns’, ‘recreation/sport/activity’, ‘lifestyle’ and ‘social and emotional’. These domains are considered important factors in measuring quality of life in patients with patellar dislocation. Although the BPII 2.0 consists of five different domains, only the mean of the 23 items is used. The total score ranges from 0 to 100, with a score of 100 indicating a good outcome [10].

### Study design

Initially, the BPII 2.0 was translated and cross‐culturally validated to a Danish version according to international guidelines of the American Association of Orthopedic Surgeons (AAOS) [3]. Secondly, the BPII 2.0 was psychometrically tested, including floor and ceiling effect, minimal detectable change (MDC), construct validity, internal consistency reliability and test‐retest reliability in a patella instability cohort. The process was conducted according with the COSMIN guidelines [21].

### Translation

Translation was performed using an accepted 5‐step stage‐divided model, as described by Beaton et al. [3], which includes forward translation, synthesis, back‐translation, expert review and pretesting.

I. Two translations from English into Danish were made independently of the original questionnaire. Both translators were bilingual, with Danish as their mother tongue language, and one had a medical background as a knee surgeon.
II. A synthesis of the two Danish translations resulted in a ‘version 1’ of the Danish questionnaire. Any discrepancies between the translations were discussed by the translators and an expert committee, and decisions were made on the final wording.
III. Two bilingual non‐medical translators with English as their mother tongue, who were ‘blind’ to the original version, made two independent back‐translations of the ‘version 1’, to check that it reflected the same content as the original version.
IV. The two back‐translations were reviewed by the original author, who commented on the differences in wording and phrasing, and made suggestions as to which best captured the essence of the questions were noted. A committee of experts consisting of three physicians with expertise in sports knee injuries reviewed the changes, resulting in a prefinal Danish ‘version 2’.
V. To test for face and content validity, the Danish ‘version 2’ was tested on 15 patients from the region of Southern Denmark with knee problems, mostly patellar instability. Subjects were then interviewed about the wording and general problems associated with the PROM. An expert committee consisting of three physicians with expertise in sports knee injuries discussed these inputs, and the changes were incorporated into the final version.

The final version, together with the results and discussions, was sent to the original author for acceptance.

### Data collection and sampling

To assess the face and content validity of the Danish version of the BPII 2.0, 15 think‐aloud interviews were conducted with patients primarily suffering from patellar instability, who were referred to Orthopedic Department at Kolding Regional Hospital. Patients with cognitive impairments that could affect their ability to respond to the PROM or who were unable to read and understand Danish were excluded from this phase.

The construct validity, internal consistency reliability and test‐retest reliability were analysed in 100 patients, aged 13–42 years, who were referred to the Orthopedic Department at Aarhus University Hospital, with test‐retest reliability specifically assessed in 57 patients. These patients were either presenting with a new patellar dislocation or returning for follow‐up after undergoing medial patellofemoral ligament (MPFL) reconstruction. These patients completed the BPII 2.0 along with the comparative instruments independently, from March 2024 to November 2024.

### Comparator instruments

#### Anterior knee pain scale (Kujala)

The Kujala is a 13‐item instrument that include limp, support, walking, stairs, squatting, running, jumping, prolonged sitting, pain, swelling, abnormal painful kneecap movements, thigh atrophy and flexion deficiency [14]. It consists of a mixture of activities, knee symptoms, sports and pain. The Kujala uses a variable Likert response format scoring from 0–5 points or 0–10. The total score ranges from 0 to 100, with 100 indicating a good outcome [14].

#### International Knee Documentation Committee (IKDC)

The IKDC has been translated and cross‐cultural validated into Danish and developed for anterior cruciate ligament patients. The questionnaire consists of 19 questions assessing knee pain, knee symptoms, activities of daily living and sports participation. The questionnaire consists of Likert scales (0–10) and categorical responses (0–4). The total score ranges from 0 to 100, with 100 indicating a good outcome [23].

#### Victorian Institute of Sports Assessment—Patella questionnaire (VISA‐P)

The VISA‐P has been developed for patients with patellar tendinopathy. The questionnaire consists of eight questions; six of which assess the level of pain (0–10), and two of which relate to participation in sport (categorical response ranges from 0 to 10 or 0 to 30). The total score ranges from 0 to 100, with 100 indicating a good outcome [25].

#### Tegner activity score (TAS)

The TAS has been translated into Danish and cross‐culturally validated in patients with various knee conditions. The TAS is a single‐item questionnaire that uses an 11‐point Likert scale (0–10) to assess the patient's level of activity and work engagement. A score of 0 indicates total disability, while a score of 10 indicates an elite level of athletic performance [13].

### Statistical analysis

All statistical analysis were performed with STATA 18 software (StataCorp, College Station). Descriptive statistics was calculated for all variables. Normal distributed continuous data were presented as mean ± standard deviation (SD), while nonnormally distributed data were presented as median and range. Categorial and binary data were presented as numbers.

#### Content validity

The ‘Version 2’ of the BPII 2.0 was face validity tested in a mixed group of 15 patients with knee problems, mainly knee instability to investigate content validity including the relevance, comprehensiveness and comprehensibility.

A semi‐structured think‐aloud interview performed by a skilled interviewer were conducted, recorded, and transcribed verbatim for each patient. The expert committee was involved in the analysis of the transcribed interviews. Changes were added, ending in a final version of the BPII 2.0.

#### Construct validity

As there is no gold‐standard for evaluating patellar dislocation, the construct validity would be analysed comparing BPII 2.0 with Kujala‐DK, IKDC, VISA‐P and TAS.

The construct validity will be assessed by testing the following hypothesis predefined and based on previous studies [4, 9, 28]. We hypothesised a correlation > 0.50 between the BPII 2.0 and the TAS, as both assess functional capacity in patients with patellar instability. Although TAS primarily measures general activity level rather than instability‐specific symptoms, we expected some degree of correlation due to the influence of patellar instability on overall activity.

1. There is a correlation (0.50 < *r* < 1.00) with the Kujala‐DK
2. There is a correlation (0.50 < *r* < 1.00) with the IKDC
3. There is a correlation (0.50 < *r* < 1.00) with the VISA‐P
4. There is a correlation (0.50 < *r *< 1.00) with the TAS

#### CFA

A CFA was conducted to assess the factor structure of the BPII 2.0‐DK. CFA was performed using a standard model with 23 items from the BPII 2.0 scale. The model was evaluated using fit indices, including the comparative fit index (CFI) and the root mean square error of approximation (RMSEA).

#### Floor and ceiling effects and MDC

Floor and ceiling effect of the BPII 2.0 are defined as a high percentage of the patients have the lowest or the highest score, respectively. Floor and ceiling effects higher than 15% are considered to be significant.

Absolute measurement errors were estimated by calculating the standard error of measurement (SEM = SD × √[1 − *r*]) [7] and converting the SEMs to the (MDC = 1.96 × √2 × SEM). The MDC defines the smallest within‐person change that can be interpreted as a ‘real’ change above the measurement error.

#### Internal consistency

Internal consistency was defined as the relatedness among the items in the questionnaire. Cronbach's Alpha was used to calculate the internal consistency of the BPII 2.0 [27]. According to internal consistency a Cronbach's Alpha > 0.70 and < 0.95 was considered a good internal consistency.

#### Test‐retest reliability

Test‐rest reliability is tested by calculating the Interclass Correlation Coefficient (ICC). Patella instability patients were asked to complete the BPII 2.0 with 6–8 days apart ensuring that they will be stable in the interim period on the construct to be measured [27]. Patients were asked to complete the questionnaires at the hospital and a prepaid envelope with BPII 2.0 and a Global Rating of Change for retesting. A text message as friendly reminder was sent to each patient 7 days after initial completeness of the PROMS.

A 17‐points global rating of change questionnaire was included ensuring that patients’ conditions have not change within the 7 days interval. Moderate change in condition were used as exclusion criteria.

The ICC with 95% confidence intervals (CI) was calculated using Pearson's test. The ICC values ranged from 0.0 to 1.0, with an ICC value above 0.70 considered acceptable for test‐retest reliability.

Furthermore, the differences between test and retest were plotted against the means to indicate whether the differences were related to the BPII 2.0 score. Test and retest differences were calculated, and systematic differences were calculated by a paired t‐test. These differences were plotted against the means of the two measures using Bland‐Altman plots with 95% CI and 95% limits of limits of agreement (LoA).

## ETHICS

The study is registered in the region's internal base of research projects. In general, information regarding subjects will be protected by the Data law of Protection and the General Data Protection Regulation will be complied with in the study. This type of study did not require approval from the Regional Ethics Committee.

Patients received verbal information about the study, emphasising that participation was voluntary. Completing the questionnaires and conducting the interviews posed no risks or side effects. The estimated time commitment for each patient was approximately 30 min in total.

## RESULTS

### Demographics

This study involved 115 patients. Fifteen patients were involved in the process of establishing the face and content validity of the questionnaire. These patients were primary patellar instability patients and few patients with other knee problems, in order to assess the impact of different conditions on the questionnaire. A total of 100 patients with patellar instability were included in the psychometric analysis. The median age was 19 years, ranging from 13 to 42 years, and 33% of patients were male. Of the 100 patients, 25 patients were referred to the orthopedic department with patella instability, 35 patients were seen at 12 months follow‐up after MPFL‐reconstruction and the remaining 40 patients were referred to their 24 months follow‐up after MPFL‐reconstruction (Table 1). All items of the BPII 2.0 and the comparing instruments were completed by all patients.

**Table 1:** Patient demographic.

	*n *= 100
Sex	
Male (*n*)	32
Female (*n*)	68
Age, year	
Median (range)	19 (13–42)
Patient MPFL‐status (*n*)	
Before MPFL reconstruction	25
≤12 months after MPFL reconstruction	35
>24 months after MPFL reconstruction	40

Abbreviations: MPFL, medial patellofemoral ligament; *n*, numbers.

#### Cross‐cultural validation and content validity of the BPII 2.0

Only minor differences were observed between the first and second Danish translations. The back‐translated versions were compared to the original BPII 2.0 version and reviewed by the expert group, resulting in no necessary adjustments.

During the face validity evaluation, only minor differences were found about the clarity of items 4 and 17. The terms ‘loss of motion’ and ‘enjoyment of life' were difficult to understand, and the items were rewritten. Similarly, the phrase ‘patellofemoral instability’ in the title was found to be difficult to understand as this is phrase is not a Danish terminology. Overall, all interviewed patients found the items of the BPII 2.0 to be relevant and comprehensible.

The developer accepted the BPII 2.0 without any modifications.

##### Construct validity

Three out of four hypotheses were confirmed with a correlation coefficient (*r*) greater than 0.50. A high correlation was observed between BPII 2.0 and Kujala‐DK (*r* = 0.73), BPII 2.0 and IKDC (*r* = 0.81) and BPII and VISA‐P (*r* = 0.67). However, the hypothesis that the correlation coefficient between BPII 2.0 and TAS would be greater than 0.50 was rejected, as the observed correlation was *r* = 0.44 (Table 2).

**Table 2:** Psychometric properties of the Danish version of the BPII 2.0.

	*r*
Construct validity	
− Kujala	0.73
− IKDC	0.81
− VISA‐P	0.67
− TAS	0.44

	Value
Reliability	
− Internal consistency (Cronbach's alpha)	0.95
− Test‐retest (ICC) (95% CI)	0.90 (0.86–0.94)
Minimal detectable change	
SEM (SD × √[1 − *r*])	7.8
MDC (1.96 × √2 × SEM)	15.3

Abbreviations: CI, confidence intervals; ICC, intraclass correlation coefficient; IKDC, International Knee Documentation Committee Subjective Knee Form; MDC, minimal detectable change; R, Pearson correlation coefficient; SEM, standard error of measurement; SD, standard deviation; TAS, Tegner activity scale; VISA‐P, Victorian Institute of Sport Assessment—Patella.

##### CFA

The CFA supported an overall unidimensional structure of the Danish BPII 2.0. The model demonstrated an acceptable fit (CFI = 0.76, RMSEA = 0.13). Standardised factor loadings ranged from 0.45 to 0.84, with most items showing moderate to strong associations with the latent construct. Items 3 and 9 exhibited weaker loadings of 0.45 and 0.48, respectively (Table 3).

**Table 3:** Confirmatory factor analysis.

	BPII 2.0 scores (*n* = 100)
Item	CFA—loadings
1	0.58
2	0.67
3	0.45
4	0.60
5	0.73
6	0.72
7	0.68
8	0.69
9	0.48
10	0.52
11	0.77
12	0.68
13	0.73
14	0.80
15	0.75
16	0.75
17	0.79
18	0.84
19	0.81
20	0.59
21	0.59
22	0.68
23	0.59
Total CFI fit	CFI: 0.76, RMSEA: 0.13

Abbreviations: BPII, Banff Patella Instability instrument; CFA, confirmatory factor analysis; CFI, comparative fit index; N, number of patients; RMSEA, root mean square error of approximation.

##### Floor and ceiling effects and MDC of the BPII 2.0

**Figure 1:**
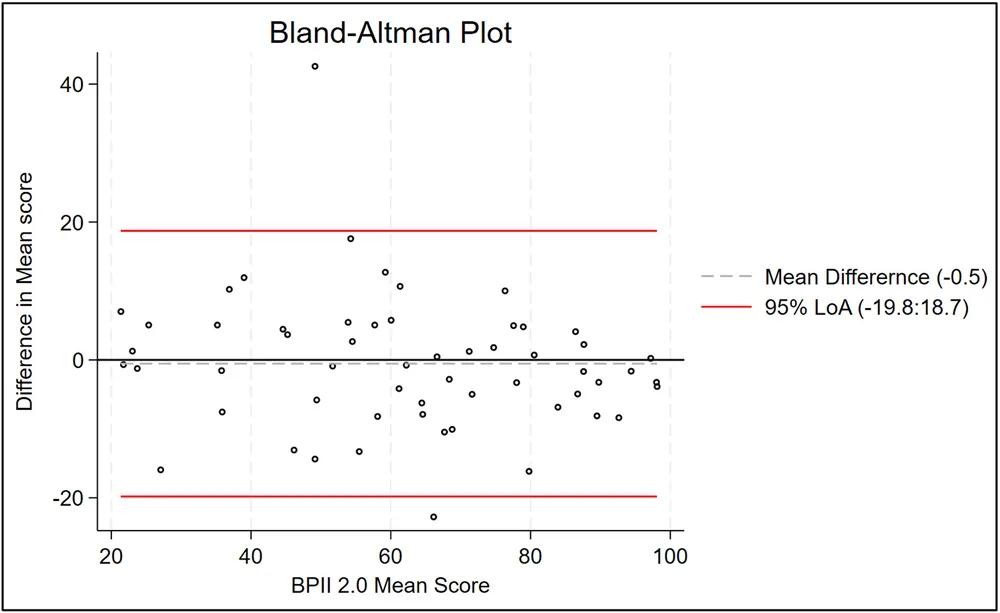
Bland–Altman plot. BPII 2.0, Banff Patella Instability Instrument; LoA, limits of agreement.

There were no ceiling or floor effects for the total score since none of the patients scored 0 or 100. The MDC of 15.3 points was calculated, indicating the smallest possible ‘real’ change in an individual's score. Figure 1 shows that the Bland–Altman plot revealed a 95% LoA ranging from −19.8 to 18.7 points. The plot also showed that the differences observed in the measurements were independent of the total score of the BPII 2.0 (Table 2).

##### Reliability

The BPII 2.0 demonstrated good internal consistency reliability, with a Cronbach's alpha of 0.95. Additionally, the ICC for test‐retest reliability was 0.90 (95% CI: 0.86–0.94) for the overall score (Table 2).

## DISCUSSION

The Danish version of the BPII 2.0 demonstrated strong psychometric properties, including high construct validity, excellent internal consistency and robust test‐retest reliability. The BPII 2.0 showed high correlations with established measures such as the Kujala‐DK, IKDC and VISA‐P, supporting its relevance and applicability for patients with patellar instability. Additionally, no ceiling or floor effects were observed, and the MDC was found to be 15.3 points, indicating that the instrument is sensitive to meaningful changes over time.

Between the first and second Danish translations, only minor differences were observed, making the synthesis process straightforward. During the cross‐cultural validation process, items 4 (loss of motion) and 17 (enjoyment of life), as well as the title, were found to be difficult to understand and had to be rewritten, as the terms used in the original version could not be translated directly into Danish. No other translated versions have presented problems with these items. The Swedish version had problems with items 6 (pivoting) and 9 (financial hardship) [29]. Item 9 may also have caused problems in the Danish version, given that the two countries have the same healthcare system, where medical interventions are funded through taxes rather than insurance.

The correlation with the Kujala‐DK (*r* = 0.73) was comparable to that reported in the Spanish (*r* = 0.74) and Arabic (*r* = 0.84) versions, and higher than the correlations observed in the Turkish (*r* = 0.53) and German (*r* = 0.58) adaptations. Correlation were all evaluated in patients with patellar dislocations. However, it was lower than the correlation of *r* = 0.98 reported for the Indonesian version, which was evaluated in patients with patellofemoral pain [1, 4, 19, 24, 30]. The lower correlation of the Turkish version compared to the Danish version may be because the Kujala‐DK and the BPII 2.0 were both validated in a Danish cohort of patients with patellar dislocation, whereas the Turkish version of the Kujala questionnaire was validated in a Turkish cohort of patients with patellofemoral pain. Differences in how the questions were translated could explain this discrepancy [30]. As the German translation and cross‐cultural adaptation of the Kujala questionnaire were first presented in 2018, the version used for construct validity in this study was considered adequate [6]. Both Indonesian versions of the BPII 2.0 and the Kujala questionnaire were developed in populations with patellofemoral pain [22, 24]. This high correlation indicates that the Indonesian version of the BPII 2.0 is useful for patients with patellofemoral pain, but does not demonstrate its suitability for patients with patellar dislocation.

This study found a higher correlation with the IKDC (*r* = 0.81) than with the Norwegian version (*r* = 0.56) [12]. This difference could be due to differences in the timing of questionnaire completion. In this study, 40% of patients completed the questionnaires 2 years after MPFL reconstruction; in the Norwegian study, all patients completed the questionnaires before or within 6 months of surgery [12].

Although no other studies have compared the BPII 2.0 and the VISA‐P, a correlation of *r* = 0.67 confirmed the hypothesis. The VISA‐P is designed for patients with patellar tendinopathy and focuses on pain and sport. In contrast, the BPII 2.0 takes a more holistic approach, evaluating physical complaints, work‐ and/or school‐related concerns, and emotional issues. Despite the differing focuses of the two instruments, the observed correlation likely reflects the shared functional limitations of knee disorders, particularly in terms of pain and activity levels. The broader scope of the BPII 2.0 may explain why the correlation is not stronger, as it captures a wider range of symptoms and life impacts beyond those addressed by the VISA‐P.

The hypothesis of *a* > 0.50 correlation between the BPII 2.0 and the TAS was based on the assumption that general activity is influenced by patellar instability. However, the observed correlation (*r* = 0.44) was lower than expected, likely due to the differing focuses of the two instruments: while the BPII 2.0 evaluates a broad range of instability‐related factors, the TAS specifically measures activity level, which may not fully capture the functional impact of patellar instability.

The CFA of the Danish version of the BPII 2.0 further supports its construct validity. To our knowledge, the factor structure of the BPII 2.0 has not previously been examined using CFA, making this study a novel contribution to the literature. The CFA results indicated a reasonable model fit (CFI = 0.76, RMSEA = 0.126), though these values suggest that the model could be improved. A higher CFI and a lower RMSEA are typically desired for a better‐fitting model. Future research with a larger, more diverse sample or adjustments to model specifications may help to achieve a more optimal fit.

As with other studies, the BPII 2.0 showed no floor or ceiling effects after MPFL reconstruction, supporting its suitability for longitudinal research ‐ even among patients assessed both before and after MPFL reconstruction, none reached the minimum (0) or maximum (100) score [1, 4, 12, 19, 24, 29, 30].

The MDC of 15.3 points in this study is comparable to the 19.7 points reported for the Norwegian version [12]. While the Norwegian version presented a higher MDC, this difference may not be substantial. The cause of this slight discrepancy remains unclear but is likely due to differences in sample characteristics or other methodological factors. Given the overall similarity between the two versions, the MDC of 15.3 points in this study indicates a ‘real’ change, though it should be interpreted with caution, as it falls within the limits of agreement (LoA) of −19.8 to 18.7 points and may be affected by measurement error. The LoA are comparable with the results from the Norwegian version (−21.1 to 16.8). Furthermore, both the Bland–Altman plots from the present study and the Norwegian study showed that the observed differences in measurements were independent of the total score of BPII 2.0 (Figure 1) [12].

Although a Cronbach's alpha of 0.95 indicates good internal consistency, which is comparable to that of the other versions [1, 4, 12, 19, 24, 29, 30], this should be interpreted with caution, as values greater than 0.90 could indicate that some items are similar, despite the fact a factor analysis has been performed when developing the BPII 2.0 version [15, 26].

The test‐retest reliability of 0.90 is comparable with the other versions ranging from 0.87 to 0.97 [1, 4, 12, 19, 24, 29, 30].

## LIMITATIONS

As there is no gold standard for assessing patellar instability, construct validity was examined by correlating the results with those of questionnaires that measure other knee problems. All of the chosen questionnaires have previously been correctly validated, are reliable and are commonly used in the Danish healthcare setting. However, the absence of clinical or radiological comparisons limits the criterion‐related validity of the BPII 2.0. Future research should consider incorporating these clinical or radiological markers to strengthen the instrument's validity.

One limitation of this study is that the potential effects of demographic factors, such as age), sex and postoperative status (i.e., follow‐up time at 12 or 24 months), on BPII 2.0 scores were not explicitly analysed. These factors could have influenced the psychometric properties of the instrument, and future studies could examine their impact on the BPII 2.0 scores to further validate its use across diverse patient subgroups. Additionally, test‐retest reliability analysis was not conducted on a sample size of 100 patients, as recommended by the COSMIN guidelines [20, 21], due to limitations in patient availability. However, this smaller sample still provided reliable results, with an ICC of 0.90. A larger sample size could have minimised the LoA and reduced the margin of error in the measurements taken at the two time points. However, the wide LoA of the within‐person change suggests that the MDC may be unusable in this setting.

Responsiveness, an important COSMIN property, was not assessed in this study. Future research should evaluate the responsiveness of the BPII 2.0, particularly in postoperative MPFL patients, by using effect size and standardised response mean to better understand the instrument's ability to detect clinically meaningful changes over time.

The collected data on psychometric properties were obtained in a single centre. If data had been collected in more centres, the scientific value of the study would have increased.

## CONCLUSION

The BPII 2.0‐DK is a relevant, comprehensible and comprehensive tool for patients with patellar instability. The CFA supports its construct validity, with a reasonable model fit. There was a high positive correlation between the BPII 2.0 and the Kujala‐DK, the IKDC and the VISA‐P. No ceiling or floor effects were observed. Furthermore, the BPII 2.0 demonstrated excellent internal consistency and high test‐retest reliability.

## AUTHOR CONTRIBUTIONS

All authors contributed to the study conception and design. Data analysis were performed by Torsten Grønbech Nielsen. Analysis and interpretation of the results was done by both authors. Pia Kjær Kristensen was a major contributor in writing the manuscript. All authors read and approved the final manuscript.

## CONFLICT OF INTEREST STATEMENT

The authors declare no conflicts of interest.

## ETHICS STATEMENT

The study was conducted in accordance with the Helsinki Declaration.

## Supporting information

Supporting Information

## Data Availability

Data are available on request.
