# Supplementary material for: Translation, cross‐cultural validity and reliability of a Danish version of the Banff Patella Instability Instrument 2.0 (BPII 2.0)
Source: J Exp Orthop. 2026 Jan 19;13(1):e70649. doi: 10.1002/jeo2.70649 (PMC12814211; doi:10.1002/jeo2.70649)
Supplement: Supplementary file 1 — Supporting Information [file JEO2-13-e70649-s001.pdf]

BANFF Patellofemoral Instabilitetsinstrument 2.0

En livskvalitetsscore for patienter med løs knæskal

Patientens navn (fornavn/efternavn): ______________________

Besøgsdato (dag/måned/år): ______________________

Din læges navn:	Hvilket knæ bliver undersøgt i dag?	Dette besøg er dit:
☐ ____________________	☐ Venstre knæ	☐ Første besøg/undersøgelse
	☐ Højre knæ	☐ Operationsdagen
	☐ Begge knæ	☐ 3 måneder efter operation
		☐ 6 måneder efter operation
		☐ 12 måneder efter operation
		☐ 24 måneder efter operation

Vejledning:

Besvar venligst hvert spørgsmål i forhold til den aktuelle status, funktion, omstændigheder og opfattelse vedrørende dit knæ som har en instabil knæskal. Vurdér de seneste 3 måneder.

Marker med en skråstreg (/) det punkt på linjen mellem 1 og 100, som bedst beskriver din situation.

For eksempel det følgende spørgsmål:

Er dette et godt spørgeskema?

0 ____ **100**

Ubrugeligt **Fantastisk**

Når skråstregen er på midten af linjen, betyder dette, at spørgeskemaet er af en middel kvalitet, eller med andre ord mellem ekstremerne “ubrugeligt“ og “fantastisk“. Det er vigtigt at sætte skråstregen ved de respektive ender af linjen, i fald ekstremerne bedst beskriver din situation.

Afsnit A: Symptomer og fysiske klager

1. Hvor besværet er du af, at din knæskal "hopper ud af led"/knæets instabilitet?

0 ______________________________ 100

Ekstremt besværet — Slet ikke besværet

2. Hvor meget smerte eller ubehag får du i knæet ved længerevarende aktivitet (over en halv time)? For eksempel når du står, går, ved sport osv.

0 ______________________________ 100

Svær smerte/ubehag — Ingen smerter overhovedet

3. Hvor meget smerte eller ubehag får du ved længerevarende siddende stilling (over en halv time)? For eksempel når du ser film, kører bil osv.

0 ______________________________ 100

Svær smerte/ubehag — Ingen smerter overhovedet

4. Har du indskrænket bevægelighed i knæet?

0 ______________________________ 100

Svær bevægeindskrænkning — Ingen bevægeindskrænkning

5. Hvor svagt føles dit knæ?

0 ______________________________ 100

Ekstremt svagt — Slet ikke svagt

Afsnit B: Arbejds- og/eller skolerelaterede problemer

**Hvis du ikke er i arbejde grundet dit knæ, skal der sættes skråstreg i yderste venstre side af linjen udfor hvert spørgsmål.

6. Hvor store problemer har du med dit knæ ved dreje-bevægelser på arbejdet og/eller skolen?

0 ______________________________ 100

Svære problemer — Slet ingen problemer

7. Hvor store problemer har du med hugsiddende stilling på arbejdet og/eller skolen?

0 ———————————————— 100

Svære problemer — Slet ingen problemer

8. Hvor stor en bekymring er det for dig at måtte sygemelde sig grundet knæproblemerne?

0 ———————————————— 100

Er en meget stor bekymring — Er slet ikke en bekymring

9. Har dine knæproblemer været skyld i økonomiske problemer for dig eller din familie?

0 ———————————————— 100

Svære økonomiske problemer — Ingen økonomiske problemer

Afsnit C: Fritid/ Sport / Aktiviteter

10. Hvor bekymret er du for, om dine fritids- og/eller sportsaktiviteter kan forværre dine knæproblemer?

0 ———————————————— 100

Ekstremt bekymret — Slet ikke bekymret

11. Er du nødt til at være forsigtig, når du deltager i fritids- og /eller sportsaktiviteter?

(Sæt en skråstreg i yderste venstre side af linjen ved 0, hvis du er ude af stand til at deltage i fritids- og/eller sportsaktiviteter på grund af din knæskade)

0 ———————————————— 100

Altid med forsigtighed — Aldrig med forsigtighed

12. Hvor bange er du for at din knæskal "hopper ud af led“, når du deltager i fritids- og/eller sportsaktiviteter?

(Sæt en skråstreg i yderste venstre side af linjen ved 0, hvis du ikke kan deltage i fritids- og/eller sportsaktiviteter, på grund af din knæskade)

0 ———————————————— 100

Ekstremt bange — Slet ikke bange

13. Hvor bekymret er du for at gå på ujævnt underlag, en våd overflade eller at gå på is?

0 ____ 100

Meget bekymret | Slet ikke bekymret

14. Kan du give dig fuldt ud i forbindelse med fritids- og/eller sportsaktiviteter?
(Sæt en skråstreg i yderste venstre side af linjen ved 0, hvis du ikke kan deltage i fritids- og/eller sportsaktiviteter på grund af din knæskade)

0 ____ 100

Aldrig muligt | Altid muligt

Afsnit D: Livsstil

15. Hvor bekymret er du for sikkerhedsproblemer på grund af din knæskade? For eksempel at gå op og ned af trapper, køre bil, at løfte små børn osv.

0 ____ 100

Svært bekymret | Slet ikke bekymret

16. I hvilket omfang er din evne til at dyrke motion og vedligeholde kondition blevet hæmmet af dine knæproblemer?

0 ____ 100

Fuldstændig hæmmet | Slet ikke hæmmet

17. I hvilken grad er din livskvalitet blevet påvirket af dine knæproblemer?

0 ____ 100

Svært påvirket | Slet ikke påvirket

18. Undgår du fritidsaktiviteter med familie og/eller venner grundet dine knæproblemer?

0 ____ 100

Undgår det altid | Undgår det aldrig

19. Er du nødt til at planlægge dine fritids og sociale aktiviteter mere end din familie og/eller venner på grund af dine knæproblemer?

0 ____ 100

Skal altid planlægge | Skal aldrig planlægge

Afsnit E: Socialt og følelsesmæssigt

20. Er du frustreret over ikke længere at kunne opfylde dine fritids- eller konkurrencemæssige behov på grund af dine knæproblemer?

(Sæt en skråstreg i yderste højre side af linjen ved 100, hvis du opfylder dine konkurrencemæssige behov. Sæt en skråstreg i yderste venstre side af linjen ved 0, hvis du ikke har konkurrencemæssige behov.)

0 ——————————————————— 100

Svært frustreret — Slet ikke frustreret

21. Har du haft svært ved følelsesmæssigt at håndtere dine knæproblemer?

0 ——————————————————— 100

Ekstremt svært — Slet ikke svært

22. Hvor tit er du nervøs for dit knæ?

0 ——————————————————— 100

Altid nervøs — Aldrig nervøs

23. Hvor bange er du for at komme til skade med knæet igen?

0 ——————————————————— 100

Meget bange — Slet ikke bange

Tak, fordi du udfyldte dette spørgeskema.

Oversat til dansk efter gældende rekommandationer af Simon Damgaard Petersen, marts 2019
Oversættelse og procedure godkendt af forfattergruppen.
